# A 74-Year-Old Woman With Dyspnea, Diffuse Bilateral Thickened Bronchovascular Bundle, Multiple Small Nodules, and a Thin-Walled Cavity

**DOI:** 10.1016/j.chpulm.2023.100028

**Published:** 2023-11-08

**Authors:** Tomoya Maruyama, Takashi Nishida, Takashi Ishiguro, Tetsu Kanauchi, Yoshihiko Shimizu, Noboru Takayanagi

**Affiliations:** a Department of Respiratory Medicine, Saitama Cardiovascular and Respiratory Center, Saitama, Japan; b Department of Radiology, Saitama Cardiovascular and Respiratory Center, Saitama, Japan; c Department of Pathology, Saitama Cardiovascular and Respiratory Center, Saitama, Japan

## Abstract

A 74-year-old Japanese woman sought treatment at the hospital for gradually progressive dyspnea and fatigue of 7 months’ duration and bilateral abnormalities on chest radiography. Past medical history was insignificant, and she had not begun any new medications for several years. She had no history of tobacco use, alcohol use, or exposure to chemicals or various dusts.

## Physical Examination Findings

Vital signs included body temperature of 36.5 °C, BP of 145/81 mmHg, heart rate of 70 beats/min, and oxygen saturation of 97% on ambient air. Pulmonary examination revealed diffuse fine crackles. Cardiac and neurologic examination findings were unremarkable. No skin rash or pitting edema, nor any superficial swollen lymph nodes, macroglossia, or hepatomegaly were noted.

## Diagnostic Studies

**Figure 1:**
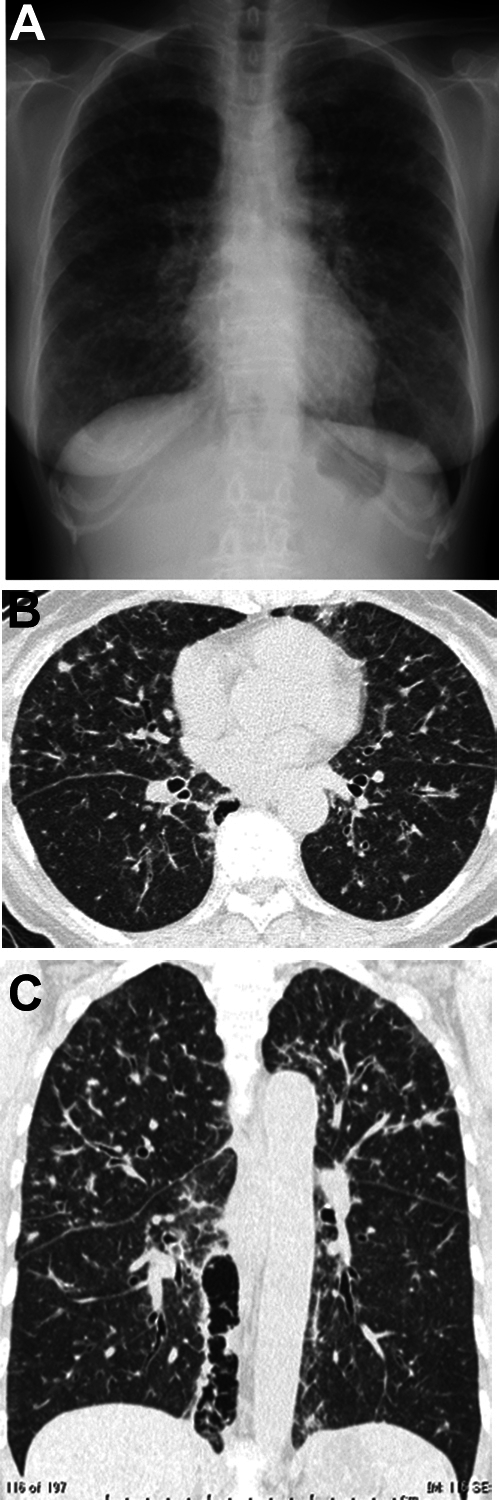
A-C, Chest imaging obtained during the patient’s first visit to the hospital. A, Chest radiographs showing diffuse ground-glass opacities and small nodules in the bilateral lung fields. B, C, Chest CT scans—axial view (B) and coronal view (C)—showing diffuse bilateral thickened bronchovascular bundles, multifocal ground-glass opacities, and small nodules with perilymphatic distribution. Interlobular septal thickening and a thin-walled cavity in the right lower lobe adjacent to the mediastinum measuring 37 × 22 × 100 mm also are present.

Pulmonary function testing showed an FVC of 2.31 L (86.5% predicted), FEV_1_ of 1.67 L (118.4% predicted), FVC to FEV_1_ ratio of 72.3%, and diffusion capacity of the lungs for carbon dioxide of 10.84 mL/min/mmHg (74.3% predicted). Laboratory data showed WBC count of 4,300/μL (neutrophils, 58.0%; lymphocytes, 30.3%; monocytes, 6.2%; and eosinophils, 4.6%), hemoglobin of 15.4 g/dL, platelets of 23.5 × 10^4^/μL, total protein of 6.5 g/dL, albumin of 4.1 g/dL, lactate dehydrogenase of 201 IU/L, C-reactive protein of 0.06 mg/dL, and Krebs von den Lungen-6 of 1,070 U/mL (reference range, < 500 U/mL), which reflect the severity of interstitial lung disease. Serum transaminase, creatine, electrolytes, and urinalysis findings were within the normal ranges, and no autoantibodies related to connective tissue diseases were detected. Chest radiography showed diffuse ground-glass opacities and small nodules in the bilateral lung fields (Fig 1A). Chest CT imaging showed diffuse bilateral thickened bronchovascular bundles, multifocal ground-glass opacities, and small nodules with perilymphatic distribution (Fig 1B). Interlobular septal thickening and a thin-walled cavity in the right lower lobe adjacent to the mediastinum measuring 37 × 22 × 100 mm also were found (Fig 1C). Chest and abdominal CT imaging showed no pleural effusion, lymphadenopathy, or hepatosplenomegaly.

**Figure 2:**
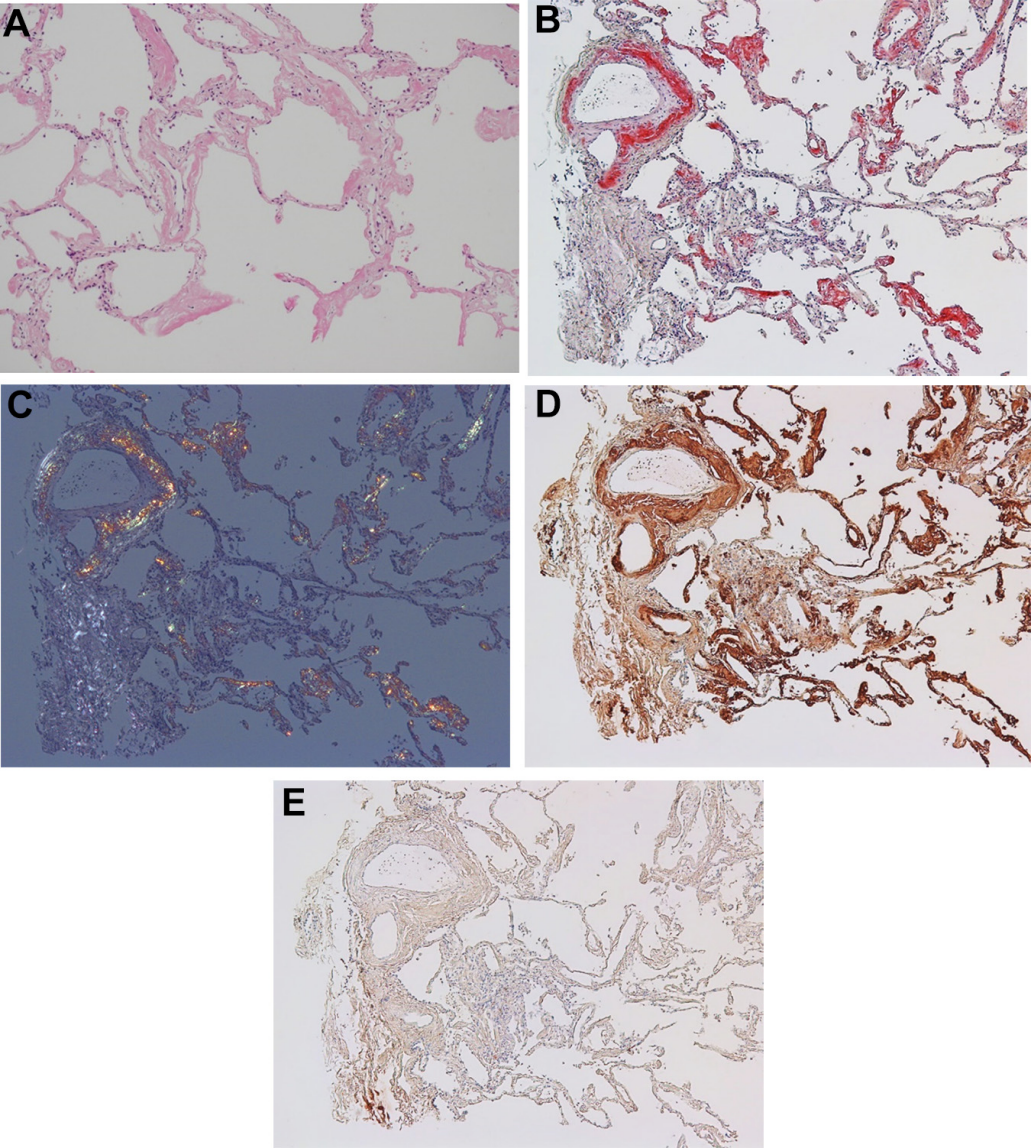
Photomicrographs showing pathologic examination results of the cryobiopsy samples. A-C, Deposition of eosinophilic materials in alveolar walls and vessels (hematoxylin-eosin stain; original magnification, × 50) (A) that show positive results for Congo-red stain (B) and green-yellow birefringence observed by polarization microscopy (C). D, E, Congo-red positive materials specifically are stained by anti-λ antibody (D) but not by anti-κ antibody (E).

**Figure 3:**
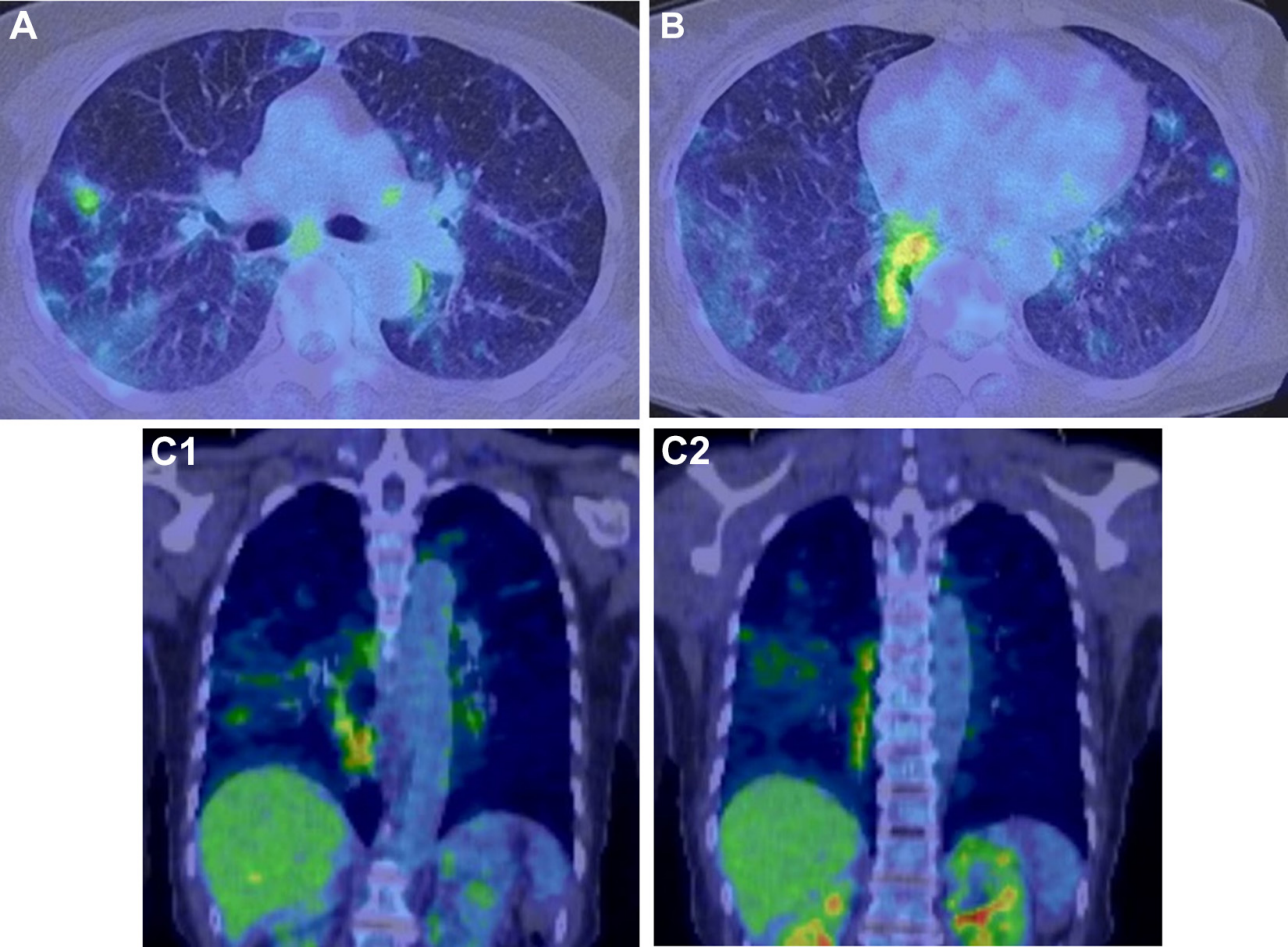
A-C, ^18^F-fluorodeoxyglucose PET-CT scans showing increased ^18^F-fluorodeoxyglucose uptake only in nodules of the bilateral lung (A) and the cavity in the right lower lobe (B, C) (maximum standardized uptake value, 4.7).

She underwent bronchoscopy with BAL and cryobiopsy. BAL fluid showed a WBC count of 1.6 × 10^4^/mL (neutrophils, 5.9%; lymphocytes, 26.1%; eosinophils, 5.9%; and macrophage, 67.7%), and the CD4 to CD8 ratio was 14.1. No significant pathogens were isolated from BAL fluid. Cryobiopsy with hematoxylin and eosin staining showed thickening of the alveolar septa and vessels by glassy eosinophilic materials (Fig 2A) stained orange-red by Congo-red staining (Fig 2B) and showing positive results for apple-green birefringence observed by polarization microscopy (Fig 2C). The eosinophilic materials specifically were stained by antibodies to λ immunoglobulin light chain (AL) (Fig 2D), but not by antibodies to κ AL (Fig 2E), serum amyloid A, transthyretin, and β2-microglobulin. Whole-body ^18^F-fluorodeoxyglucose PET-CT imaging showed increased ^18^F-fluorodeoxyglucose uptake only in nodules in the bilateral lungs and the right lower lobe cavity (maximum standardized uptake value, 4.7) (Fig 3A, 3B). Electrocardiography findings were within the normal range, and transthoracic echocardiography showed no abnormal findings. Cardiac-specific troponin and brain natriuretic peptide were not increased. Biopsies of abdominal fat, skin, bone marrow, and gastroduodenal mucosa showed no abnormalities. Additional tests showed decreased serum immunoglobulin levels (IgG, 642 mg/dL; IgA, 59 mg/dL; IgM, 22 mg/dL; and IgE, < 10 mg/dL) and increased serum free light-chain levels. The λ chain was increased significantly to 191.5 mg/L (normal, 5.7-26.3 mg/L), whereas the κ chain was 7.1 mg/L (normal, 3.3-19.4 mg/L), and the κ to λ ratio was low at 0.04 (normal range, 0.26-1.65). However, monoclonal protein or Bence-Jones protein was not detected in serum or urine by immunofixation and electrophoresis, and bone marrow biopsy showed normal morphologic features of 3.2% plasma cells, with no neoplastic change, abnormal chromosomes, or an imbalanced κ to λ ratio.

*What is the diagnosis?*

*Diagnosis:* Localized pulmonary diffuse-alveolar septal AL amyloidosis and light-chain monoclonal gammopathy of undetermined significance (MGUS)

## Discussion

Amyloidosis is caused by insoluble misfolded autologous protein and its extracellular deposition as fibrils that result in organ dysfunction. Amyloidosis is classified by disease distribution and precursor proteins. Systemic amyloidosis affects multiple organs, whereas localized amyloidosis shows limited amyloid deposition to one organ. Amyloid subtypes can be identified by immunohistochemistry or laser microdissection followed by mass spectrometry-based proteomic analysis. The number of known human amyloid fibril proteins is now 42, and AL, serum amyloid A, and transthyretin are common types of proteins (Table 1). AL amyloid, protein derived from monoclonal γ globulin, makes up monoclonal protein. Immunoglobulin comprises two heavy chains and two light chains, being either κ or λ. Imbalance of κ and λ chains often is present in monoclonal gammopathies.

AL amyloidosis can cause amyloid deposition to the urinary tract, larynx, lung, skin, eye, and other organs. Diagnosis of localized AL amyloidosis is predicated on biopsy-proven amyloid deposition in one organ and negative results for electrophoresis and immunofixation of serum and urine. If a circulating monoclonal protein is present, the light-chain isotype must be different from that of the amyloid deposits. Systemic AL amyloidosis can be ruled out comprehensively when bone marrow and fat pad biopsy examinations show no amyloid deposition.

In the respiratory system, amyloid can deposit to trachea, bronchi, mediastinal lymph nodes, pleura, and lung. Lung amyloidosis is classified into nodular and diffuse alveolar-septal types. Nodular pulmonary amyloidosis is defined as one or more tumefactive amyloid deposits involving the lungs. Diffuse alveolar-septal amyloidosis shows amyloid deposition to the alveolar septum and small vessels. The most common amyloid protein of diffuse alveolar-septal amyloidosis is AL, but cases caused by serum amyloid A, wild-type transthyretin, and variants of transthyretin amyloidosis also are reported. Most cases are of systemic amyloidosis. To our knowledge, seven unusual cases of diffuse alveolar-septal amyloidosis without evidence of systemic amyloidosis have been described.

High-resolution CT scan findings of diffuse-septal amyloidosis mainly are nodular interlobular and intralobular thickening, subpleural and peribronchovascular nodules, and diffuse, small, well-defined nodules. Less common findings include cysts or cavities, areas of ground-glass opacification, consolidation, traction bronchiectasis, and foci of calcification within nodules. ^18^F-fluorodeoxyglucose PET-CT imaging in 18 of 18 patients with pulmonary AL amyloidosis showed avidity at the sites of amyloid deposition with a median standardized uptake value of 2.7.

Transbronchial lung biopsy or cryobiopsy are useful methods to diagnose diffuse alveolar-septal amyloidosis. Larger samples can be obtained with cryobiopsy than with transbronchial lung biopsy, but some cases have been diagnosed by surgical lung biopsy, which provides the most accurate diagnosis, but is relatively invasive.

Some cases of pulmonary localized AL amyloidosis complicated by MGUS have been reported. MGUS is a premalignant, clonal plasma cell disorder characterized by the presence of monoclonal protein, < 10% clonal plasma cells in bone marrow, and absence of multiple myeloma or related lymphoplasmacytic malignancies. MGUS is present in 3% of the general population aged 50 years and older, but in only 0.3% among those younger than 50 years. Three MGUS subtypes exist: IgM MGUS, non-IgM MGUS, and light-chain MGUS. Because the precursor protein of AL amyloidosis is a light chain of immunoglobulin, detection of monoclonal protein in a patient’s serum or urine may be the clue to diagnosing AL amyloidosis (Table 2). In a case series of 581 patients with systemic AL amyloidosis, 98.1% harbored monoclonal gammopathy. However, detection of monoclonal protein is less frequent in cases of localized AL amyloidosis: 121 of 606 patients (20%) showed monoclonal protein and 27 of 413 patients (7%) showed abnormal serum free light-chain levels. The mechanism underlying the high MGUS prevalence in localized AL amyloidosis remains to be elucidated.

Treatment of systemic AL amyloidosis is based on various antiplasma cell therapies targeting underlying plasma cell clones. Although survival has improved over past decades, the prognosis of systemic AL amyloidosis remains poor, with a median overall survival of about 4 years and a reported average survival of patients with systemic amyloidosis who show pulmonary involvement of about 16 months. Treatment of localized amyloidosis is guided by patient severity and mostly is treated by endoscopic or surgical removal of the amyloid and, less frequently, radiotherapy. No established therapies exist for pulmonary localized amyloidosis, and observation often is selected if a patient’s symptoms and conditions are stable. Solitary nodule types are treated with resection of the lesion, and diffuse alveolar types occasionally are treated with chemotherapy. The estimated 5-year overall survival of patients with pulmonary localized AL amyloidosis is reported to be good at approximately 90%. However, these rates are reported for only seven patients with localized diffuse alveolar-septal AL amyloidosis, and it is difficult to predict the prognosis of this condition because of its rarity. Careful monitoring of the patient’s general condition, chest imaging findings, and laboratory data are required.

### Clinical Course

Although most cases of diffuse alveolar-septal amyloidosis are of systemic amyloidosis, the present patient’s diagnosis was localized AL amyloidosis. Because of the low immunoglobulin level and κ to λ ratio in this patient, we considered the possibility of plasma cell neoplasm. However, neither monoclonal protein nor Bence-Jones protein was detected in serum and urine, and no increases of plasma cells and amyloid deposition were found in the bone marrow. Moreover, amyloid deposition was not found in biopsied specimens of abdominal fat, skin, and gastroduodenal mucosa, which met the criteria of localized AL amyloidosis and light-chain MGUS. We decided not to treat the patient with antiplasma cell therapy and to observe her carefully. After 9 months, no remarkable changes were found in subjective symptoms, chest imaging findings, immunoglobulin values, and serum free light-chain levels.

## Clinical Pearls

1. *Diffuse alveolar-septal amyloidosis is characterized by amyloid deposition to the alveolar septum and small vessels and mostly presents as systemic amyloidosis.*
2. *Diffuse alveolar-septal amyloidosis should be considered in the differential diagnosis of patients with peribronchovascular interstitial thickening and nodules with perilymphatic distribution with or without cysts or cavities.*
3. *The diagnosis of localized diffuse alveolar-septal amyloidosis requires lung biopsy, and cryobiopsy also may be useful.*
4. *Localized pulmonary diffuse alveolar-septal amyloidosis is a rare form with no established therapy.*

## Funding/Support

The authors have reported to *CHEST Pulmonary* that no funding was received for this study.

## Uncited

**Table 1:** Common Types of Amyloidosis

Type of Amyloidosis	Precursor Protein	Target Organs
Systemic AL	Light chain	All organs
Localized AL	Light chain	Urinary tract, larynx, lung, skin, eye
Systemic hereditary transthyretin	Variant type of transthyretin	Heart, peripheral nervous system
Systemic wild-type transthyretin	Wild type of transthyretin	Heart
Systemic AA	Serum amyloid A	Kidneys

AA = serum amyloid A; AL = immunoglobulin light chain.

**Table 2:** Distinction of AL Amyloidosis and Light-Chain MGUS

AL amyloidosis	• In diagnosis of AL amyloidosis, it is required to prove deposition of AL amyloid in organ tissues. • Monoclonal protein or urine Bence-Jones protein sometimes show positive results; however, it is not essential to make diagnosis.
Light-chain MGUS	All criteria must be met: • Abnormal FLC ratio • Increased level of involved light chain • No immunoglobulin heavy-chain expression on immunofixation • Absence of end-organ damage that can be attributed to the plasma cell proliferative disorder • Clonal bone marrow plasma cells < 10% • Urinary monoclonal protein < 500 mg per 24 h

AL = immunoglobulin light chain; MGUS = monoclonal gammopathy of unknown significance; FLC = free light chain.

## Financial/Nonfinancial Disclosures

None declared.
